# Supplementary material for: Comparison between Bilateral Ultrasound-Guided Quadratus Lumborum Block and Sacrococcygeal Epidural in Cats Undergoing Ovariectomy
Source: Vet Sci. 2024 Jan 8;11(1):25. doi: 10.3390/vetsci11010025 (PMC10819764; doi:10.3390/vetsci11010025)
Supplement: Supplementary file 1 [file vetsci-11-00025-s001.zip › vetsci-2747460-supplementary.pdf]

SUPPLEMENTARY FILE

This supplementary file aims to clarify the methods employed in calculating sample size and effect size. The goal is to make these procedures easily comprehensible for the readers. Calculating the effect size a priori presents challenges, particularly when past data is unavailable. As far as our knowledge extends, no studies have outlined the effect size of the hypotension variable when comparing sacrococcygeal epidural versus ultrasound-guided quadratus lumborum block. Consequently, we gathered preliminary data to estimate the effect size and facilitate the calculation of sample size (Table S1).

Table S1. The percentage of hypotension detected in each group during the preliminary study. QLB, Ultrasound-guided quadratus lumborum block group with bupivacaine 0.25%; ScE, Sacrococcygeal epidural with bupivacaine 0.25%.

	n	QLB	ScE
Hypotension	6/20	1/10 (10%)	5/10 (50%)

In this preliminary analysis, Phi Coefficient or Cramer's V was computed using SPSS version 26 (IBM SPSS Statistics; IBM Corp., NY, USA)(Table S2).

Table S2. Phi Coefficient and Cramer´s V values.

	Value	*Significance*
Phi Coefficient	-0. 436	0.05
Cramer´s V	0. 436	0.05

Based on the results presented in table S2, it can be inferred that the effect on the incidence of hypotension lies between a medium (0.3) and a large effect (0.5). This information was instrumental in estimating the effect size used to determine the sample size for our experiment, utilizing G*Power 3.1.9.7 (Figure S1). We determined a minimum sample size of 50 cats, taking into account an estimated effect size of 0.4, an alpha error of 0.05, and a power of 0.8.

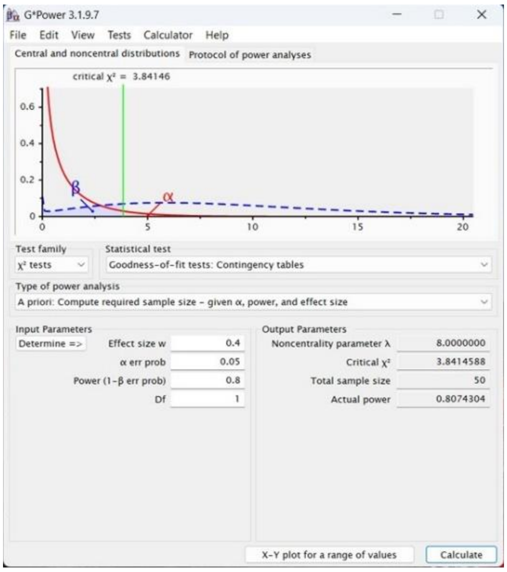

Figure S1. Total sample size calculation using G*Power 3.1.9.7.
